# P-1503. Dual β-Lactam Approach for Treatment of *Mycobacterium abscessus* Infections in Adults – Results in 19 Patients

**DOI:** 10.1093/ofid/ofae631.1672

**Published:** 2025-01-29

**Authors:** Gautam Kalyatanda, Khalid M Dousa, Hannah Lyons, Markos Mardourian, Jackson Rhodes, Alexandria Iakovidis, Guy El-Helou, Brigid Wilson, Charles L Daley, Steven M Holland, Barry N Kreiswirth, David C Nguyen, Eunjeong Shin, Sebastian G Kurz, John L Johnson, Robert A Bonomo

**Affiliations:** University of Florida, Gainesville, Florida; Louis Stokes Cleveland VA Medical Center, Cleveland, Ohio; University of Florida, Gainesville, Florida; University of Florida, Collage of Medicine, Gainesville, Florida; 3University of Florida, Collage of Medicine, Gainesville, Florida; 3University of Florida, Collage of Medicine, Gainesville, Florida; University of Florida, Collage of Medicine, Gainesville, Florida; VA Northeast Ohio Healthcare System, Cleveland, Ohio; National Jewish Health, Denver , CO; National Institutes of Health, Bethesda, Maryland; Center for Discovery and Innovation, Hakensack Meridian Health, Nutley, New Jersey; Rush University Medical center, Chicago, Illinois; Case Western Reserve University, Lakewood, Ohio; University Hospitals of Tuebingen, Tuebingen, Baden-Wurttemberg, Germany; Division of Infectious Diseases & HIV Medicine in the Department of Medicine, Case Western Reserve University School of Medicine, Cleveland, Ohio; Case Western Reserve University/ Louis Stokes Cleveland VA Medical Center, Cleveland, OH

## Abstract

**Background:**

The prevalence of *Mycobacterium abscessus* (*Mab*) infection is rising, with immunocompromised individuals being especially susceptible. While treatment success for the subspecies *abscessus* has been as low as 33%, recent studies and case reports suggest potential efficacy of select dual β-lactam (Dβ). This study evaluated the efficacy and tolerability of select Dβ in treating MDR-*Mab* infections, guided by *in-vitro* susceptibility results.

**Figure:**
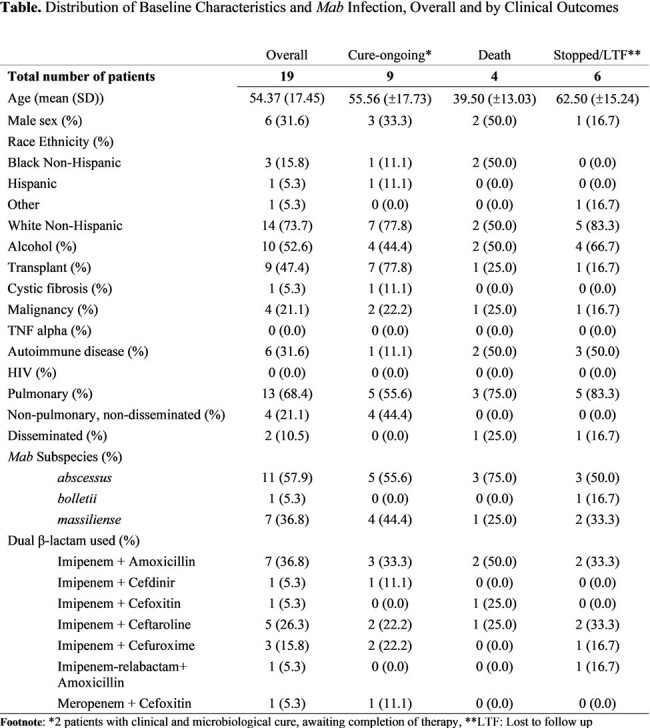

**Methods:**

We reviewed the treatment of 19 adults with *Mab* infection at the University of Florida Hospital, Gainesville, who underwent Dβ synergy testing between January 2019 and March 2023. Medical histories, laboratory results, and clinical outcomes were reviewed. We summarized patient and infection characteristics and compared the time from diagnosis to Dβs initiation across outcomes

**Figure:**
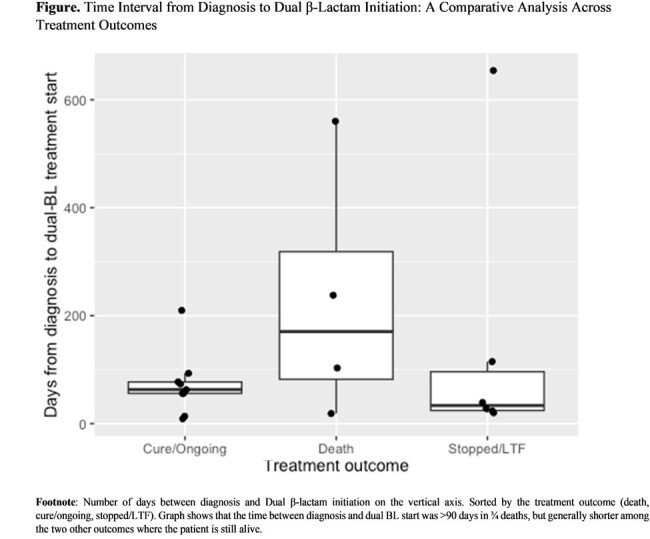

**Results:**

Of the 19 patients (mean age: 54 years; 68% females), 11 (58%) had undergone solid organ transplantation or had cancer, and 6 (32%) were on immunosuppressants for autoimmune diseases. Pulmonary infections predominated (68%), with 47% of isolates were macrolide resistant. 11 patients (58%) were due to *subspecies abscessus*. The Dβs most frequently used for treatment were imipenem combined with either amoxicillin (31%) or ceftaroline (26%). Successful clinical cure or stable culture conversion with ongoing treatment was observed in 9 (47%) cases. 4 patients died during treatment. We observed a trend towards shorter times before initiation of Dβ treatment among patients who were cured. Time to initiation of Dβ therapy exceeded 90 days in 3 of 4 patients who died. Eighteen of 19 patients tolerated dual β-lactams therapy well. Among the 5 who discontinued treatment, the multi-drug regimen, not specifically the dual β-lactams, was the likely cause

**Conclusion:**

Treatment with Dβ-containing regimens was associated with higher cure rates in this cohort of severely ill, immunocompromised patients. Earlier treatment correlated with better outcomes. Given the poor response to current treatment regimens and the initial promising results of Dβ therapy, clinical trials are planned. Finally, the chosen Dβs may not fully represent the optimal selection due to patient-driven preferences, possibly underscoring a conservative rather than optimal treatment outcome.

**Disclosures:**

**All Authors**: No reported disclosures

